# High sodium intake does not worsen low potassium‐induced kidney damage

**DOI:** 10.14814/phy2.15671

**Published:** 2023-04-20

**Authors:** Yahua Zhang, Juan Pablo Arroyo, Fabian Bock, Ming‐Zhi Zhang, Raymond C. Harris, Andrew S. Terker

**Affiliations:** ^1^ Division of Nephrology, Department of Medicine Vanderbilt University Medical Center Nashville Tennessee USA; ^2^ Vanderbilt Center for Kidney Disease Nashville Tennessee USA; ^3^ Department of Veterans Affairs Tennessee Valley Healthcare System Nashville Tennessee USA

**Keywords:** injury, kidney, potassium, sodium

## Abstract

High sodium and low potassium intake have both been linked to poor cardiovascular health outcomes and increased mortality rates. A combination of the two is thought to be particularly detrimental. While mechanisms are multiple, the kidney is an important target of harmful effects and low potassium influences on both proximal and distal nephron segments are especially potent. We recently reported that a combined high sodium/low potassium diet causes kidney injury and that low potassium in isolation can have similar effects. However, how sodium intake alters this process is not well‐understood. Here we tested the hypothesis that a high sodium intake amplifies effects of low dietary potassium on kidney injury. We observed adding high sodium to low potassium caused an expected increase in blood pressure, but did not worsen markers of kidney injury, inflammation, and fibrosis. It also did not increase abundance or phosphorylation of the sodium chloride cotransporter or its regulatory kinases, SPAK and OxSR1, known renal targets of low potassium. Findings support the claim that dietary potassium deficiency, and not high sodium, is a dominant factor affecting kidney injury in animal models of high sodium/low potassium intake. This suggests further investigation is required to identify optimal ranges of sodium and potassium intake in both healthy populations and in those with kidney disease.

## Graphical abstract

Adding high sodium to a low‐potassium diet caused an expected increase in blood pressure, but did not worsen markers of kidney injury, inflammation, and fibrosis. It also did not increase the abundance or phosphorylation of the sodium chloride cotransporter or its regulatory kinases, SPAK and OxSR1, known renal targets of low potassium. Findings support the claim that dietary potassium deficiency, and not high sodium, is a dominant factor affecting kidney injury in animal models of high sodium/low potassium intake.

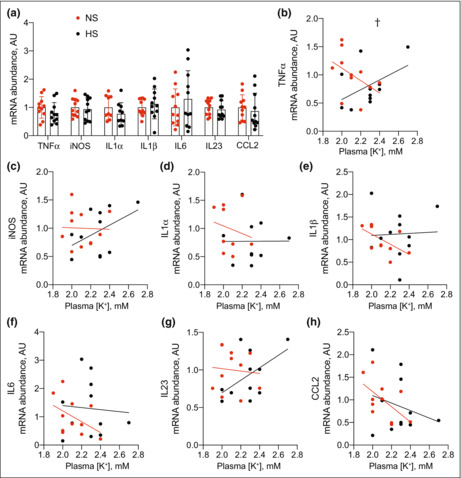

## 1 INTRODUCTION

Dietary micronutrient content strongly influences cardiovascular health. Sodium (Na^+^) and potassium (K^+^) intake are especially important as demonstrated by multiple clinical and epidemiological studies. These reports have identified correlations between both high Na^+^ and low K^+^ intake and negative outcomes, including hypertension, stroke, chronic kidney disease (CKD), and mortality (Elfassy et al., 2020; Mente et al., 2014; Neal et al., 2021; O'Donnell et al., 2014; Xi et al., 2015; Yang et al., 2011). The Na^+^‐to‐K^+^ ratio, which is high in most modern diets, has often been associated with poor outcomes better than either parameter alone suggesting an interaction between the two variables (Vaudin et al., 2022; Yang et al., 2011).

Investigators have identified multiple pathways by which high Na^+^ exerts deleterious effects on blood pressure and end organs. Several systems have been implicated in the pathogenesis including the kidneys (Terker et al., 2015), vasculature (Boegehold, 2013), immune system (Guzik et al., 2007), nervous system (Adrogue & Madias, 2007), and skin (Machnik et al., 2009). While Na^+^ had previously received more attention, recent work has focused on determining how low K^+^ intake is harmful and if the two factors might synergize to amplify negative effects.

Multiple organ systems have also been hypothesized as mediators of low K^+^ on cardiovascular health (Adrogue & Madias, 2007). Strong evidence has emerged for direct effects of low K^+^ intake on the kidneys, with influences on both proximal and distal nephron segments. The distal convoluted tubule is now a well‐accepted target, increasing apical Na^+^ reabsorption via the NaCl cotransporter (NCC) in the presence of reduced extracellular K^+^ (Terker et al., 2015). This effect provides an explanation for the long‐standing observation that low K^+^ raises blood pressure (Addison, 1928). Effects on proximal cell physiology have also been known for some time, including increased ammoniagenesis, gluconeogenesis, and Na^+^ reabsorption (Boyd‐Shiwarski et al., 2020; Kamm & Strope, 1973; Tannen, 1977), and we recently reported that a low K^+^ diet causes proximal epithelial injury, inflammation, and fibrosis (Terker et al., 2022).

Because modern diets contain both high Na^+^ and low K^+^ content, our studies, and those from other groups, often test effects of a combined high salt/low K^+^ diet to model the dietary profile consumed by most individuals (Boyd‐Shiwarski et al., 2020; Maeoka et al., 2022; Terker et al., 2015; Vitzthum et al., 2014). However, low K^+^ intake alone is sufficient to affect proximal and distal nephron segments and cause kidney injury (Terker et al., 2022; Vallon et al., 2009). The addition of high Na^+^ in the presence of low K^+^ raises blood pressure (Boyd‐Shiwarski et al., 2020; Terker et al., 2015; Vitzthum et al., 2014) and can further reduce blood K^+^ by increasing Na^+^/K^+^ exchange in principal cells (Young, 1985; Young et al., 1984), but whether the addition of high Na^+^ independently worsens kidney inflammation and injury in low K^+^ models remains unknown.

Here we tested the hypothesis that high Na^+^ intake amplifies effects of low dietary K^+^ on kidney injury. We observed that the addition of high Na^+^ to a low K^+^ diet caused an expected increase in blood pressure, but effects on kidney injury, inflammation, and fibrosis were not worsened compared to low K^+^ alone. The findings support the claim that dietary K^+^ deficiency, and not high Na^+^, is a dominant factor affecting kidney injury in animal models of high Na^+^/low K^+^ intake. This suggests further investigation is required to identify optimal ranges of Na^+^ and K^+^ intake in both healthy populations and those with CKD.

## 2 METHODS

### 2.1 Animals

All animal experiments were performed in accordance with the guidelines and with the approval of the Institutional Animal Care and Use Committee of Vanderbilt University Medical Center. Male C57Bl/6 animals aged 8–10 weeks were used for all experiments.

### 2.2 Diets

Normal Na^+^, K^+^‐deficient diets (TD.88239, 15–30 ppm [0.0015%–0.003%] K^+^, 0.3% Na^+^, and 0.45% Cl^−^, 0.06% Ca^2+^) and high Na^+^, 0.04% K^+^ diets (TD.210231 0.04% K^+^, 2.36% Na^+^, 3.65% Cl^−^ 0.1%, Ca^2+^) diets were purchased from Envigo. Animals were treated for 3 weeks. Contents are also listed in Table 1.

**TABLE 1:** Micronutrient content (% weight) of the normal salt (NS) and high salt (HS) diets used.

Diet	K, %	Na, %	Cl, %	Ca, %
NS	0.0015–0.003	0.3	0.45	0.06
HS	0.04	2.36	3.65	0.1

### 2.3 Urine electrolytes

During the third week of treatment morning spot urines were collected and urine Na^+^ and K^+^ were measured using a Diamond Diagnostics Carelyte Plus unit. Urine calcium was measured using the o‐cresolphthalein complexone method (Pointe Scientific). Data were normalized to urine creatinine measured with The Creatinine Companion kit (Exocell).

### 2.4 Blood electrolytes

Blood electrolytes were measured on samples obtained by cardiac puncture with an iSTAT analyzer using Chem8+ cartridges (Abbott) or with a Diamond Diagnostics Carelyte Plus unit.

### 2.5 Blood pressure measurements

Blood pressure was measured using a BP‐2000 Series II blood pressure analysis system (Visitech systems). Animals were trained on the machine for at least 3 days during which data was not recorded. Following this training period, blood pressure was then measured for at least 3 days during the third week of dietary treatment.

### 2.6 Western blot

Kidneys were snap frozen in liquid nitrogen at the time of euthanasia and subsequently transferred to −80°C for storage. For tissue lysate preparation, tissue was homogenized with a Tissue‐Tearor homogenizer (Biospec Products), in lysis buffer as previously reported (Sasaki et al., 2021). Lysate was then centrifuged at 6000 rpm for 15 min. Protein concentration was measured by BCA protein assay (Pierce) followed by gel electrophoresis on a 4%–20% Bis‐Tris gel (Bio‐Rad).

### 2.7 Quantitative PCR

PCR was performed as described previously (Terker et al., 2022). Kidneys were snap frozen in Trizol reagent (Invitrogen) at the time of euthanasia and RNA was subsequently isolated according to the manufacturer's protocol. SuperScript IV First‐Strand Synthesis System kit (Invitrogen) was used to synthesize cDNA from equal amounts of total RNA from each sample. Quantitative RT‐PCR was performed using TaqMan real‐time PCR (7900HT; Applied Biosystems). The Master Mix and all gene probes were purchased from Applied Biosystems. The following Thermo Fisher probes were used: TNFα (Mm99999068), iNOS (Mm00440502), IL1α (Mm00439621), IL1β (Mm00434228), IL6 (Mm00446190), IL23 (Mm00518984), CCL2 (Mm00441242), Kim1 (Mm00506686), NGAL (Mm01324470), Col1α1 (Mm00801666), Col3α1 (Mm01254476), FN (Mm01256744), αSMA (Mm01546133). Relative quantification of specific PCR products was determined by the 2^−ΔΔCT^ method. Data were normalized to RPS18 (Mm02601777).

### 2.8 Antibodies

Antibodies used for Western blot: pNCC‐T46 (1:1000; MRC at the Univ of Dundee), total NCC (1:10,000; Bostanjoglo et al., 1998), total SPAK (1:5000; McCormick et al., 2011), total OxSR1 (1:5000; Terker et al., 2014), pSPAK/pOxSR1 (1:1000; Millipore Sigma 07‐2273), beta‐actin (1:5000; Millipore A1978).

### 2.9 Statistical analysis

Data are presented as mean ± SD. Comparisons were made using an unpaired Student's *t*‐test. Bonferroni correction was used as indicated to correct for multiple comparisons.

## 3 RESULTS

We have shown that effects of low dietary K^+^ on the kidney are mediated by reductions in blood K^+^ (Terker et al., 2015, 2022). Here we sought to investigate effects of high dietary Na^+^ in two groups of low K^+^‐fed animals with similar blood K^+^ levels. However, it is known that increased distal Na^+^ delivery to principal cells promotes K^+^ secretion along the connecting tubule (Young, 1985; Young et al., 1984), lowering blood K^+^. Consistent with this, our recently reported blood K^+^ levels in mice consuming a normal salt (NS), K^+^‐deficient (0%) diet were similar to those in mice consuming a high salt (HS), 0.04% K^+^ diet (NS: 2.4 ± 0.06 mM vs. HS: 2.6 ± 0.06 mM) (Terker et al., 2022). Further reductions in dietary K^+^ in the setting of HS led to even lower blood K^+^ levels. We did not want a lower blood K^+^ in HS‐fed animals, due to greater distal Na^+^ delivery and enhanced K^+^ secretion, to confound our results. Therefore, in an attempt to match blood K^+^ levels in two groups of animals we supplemented a group of HS‐fed mice with 0.04% dietary K^+^ while maintaining another group of normal NS‐fed mice on a K^+^‐deficient diet (Table 1).

**FIGURE 1:**
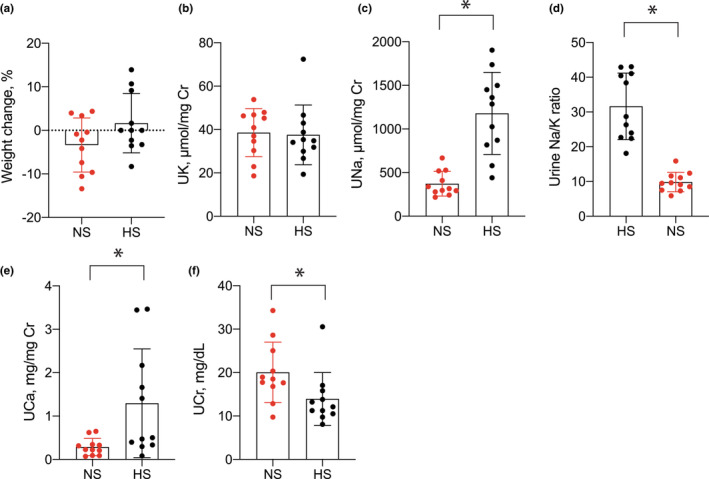
Urine electrolytes in mice consuming NS and HS diets. (a) Weight change (%) over the 3‐week study period for animals consuming NS and HS diets. (b) Urine K^+^, (c) urine Na^+^, (d) urine Na^+^‐to‐K^+^ ratio, (e) urine Ca^2+^, and (f) urine creatinine during the third week of treatment in animals consuming NS or HS diets. *N* = 11 per group. *p < 0.05 by unpaired *t*‐test. HS, high salt; NS, normal salt.

We used these two groups to test effects of HS intake in the setting of low dietary K^+^. Animals were treated for 3 weeks and differences in weight change between groups were not observed during the treatment period (Figure 1a). In the third week of treatment, we measured urine K^+^ excretion and observed nearly identical values between groups (Figure 1b). As expected, urine Na^+^ was significantly greater in the HS‐fed animals as was the urine Na^+^‐to‐K^+^ ratio (Figure 1c,d). Because a high salt/low K^+^ diet has been shown to increase urine calcium (Ca^2+^) excretion and promote kidney stone formation (Terker et al., 2015; van der Wijst et al., 2018) we also measured urine Ca^2+^, which was observed to be greater in the presence of HS intake (Figure 1e). The urine creatinine concentration was slightly lower in HS‐fed mice (Figure 1f).

**FIGURE 2:**
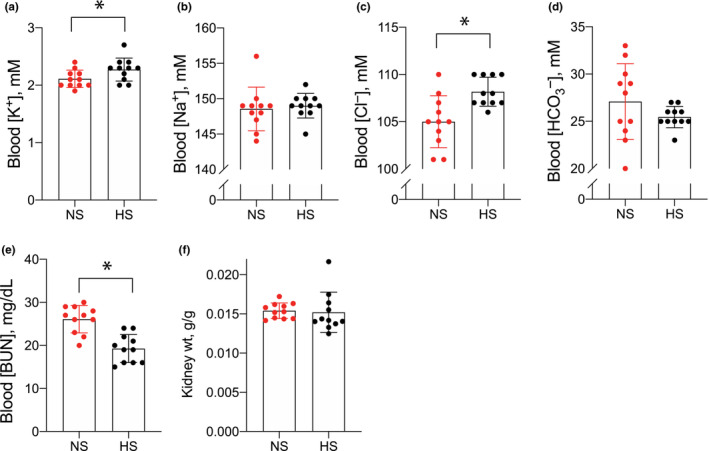
Blood electrolytes in mice consuming NS and HS diets. (a) Blood K^+^, (b) blood Na^+^, (c) blood Cl^−^, (d) blood HCO_3_^−^, (e) blood [BUN], and (f) kidney weight in animals consuming NS or HS diets. *N* = 11 per group. *p < 0.05 by unpaired *t*‐test. HS, high salt; NS, normal salt.

When comparing blood electrolytes, we found that mean blood K^+^ values were similar (NS: 2.1 ± 0.05 mM vs. HS: 2.3 ± 0.06 mM). Because the study was powered to detect small changes, observed differences did reach the threshold for statistical significance (Figure 2a). Differences in blood Na^+^ and HCO_3_^−^ were not detected, though Cl^−^ was slightly higher and BUN was slightly lower in the HS group (Figure 2b–e). Kidney weight was also not different between groups (Figure 2f). All animals had blood creatinine concentrations <0.2 mg/dL, which were all below the limit of detection using our method of analysis.

**FIGURE 3:**
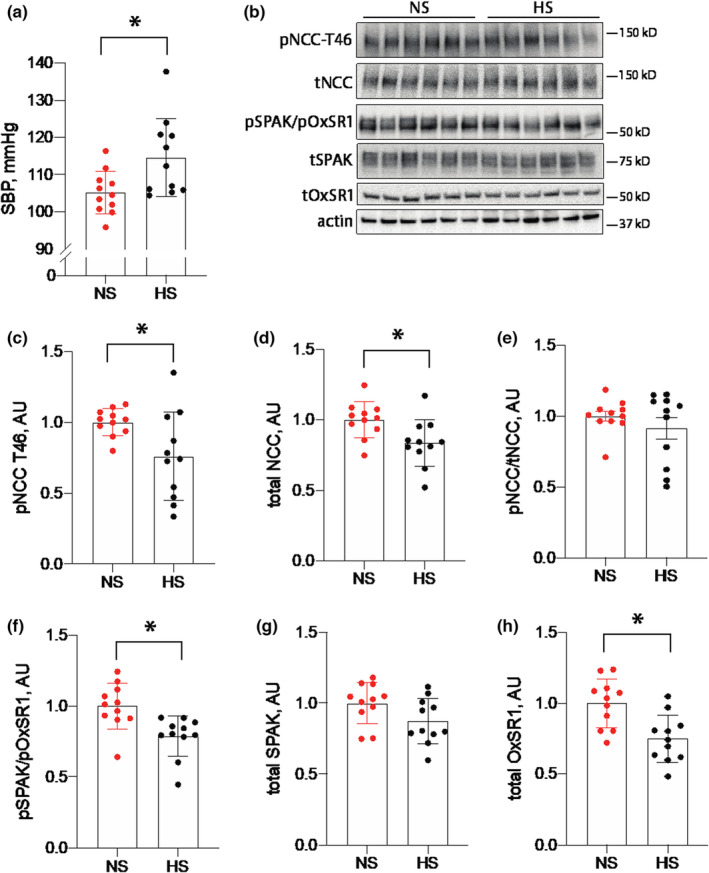
Blood pressure and SPAK/OxSR1/NCC abundances in mice‐consuming NS and HS diets. (a) Systolic blood pressures during the third week of treatment from animals consuming NS or HS diets. (b) Representative Western blot images of pNCC, tNCC, pSPAK/pOxSR1, tSPAK, and tOxSR1 from animals consuming NS or HS diets. (c–h) Actin‐normalized quantification for each protein individually and the pNCC‐to‐total NCC ratio. *N* = 11 per group. *p < 0.05 by unpaired *t*‐test. HS, high salt; NCC, NaCl cotransporter; NS, normal salt; pNCC, phosphorylated NCC.

Low K^+^ intake is recognized to cause salt sensitivity in blood pressure. As higher blood pressure can cause kidney injury we measured blood pressure in both groups of animals during the third week of the study. Consistent with previous reports, HS intake increased systolic blood pressure in the presence of low K^+^ intake when compared with the NS control group (Figure 3a). Low K^+^ stimulation of NCC is thought to be a major contributor to this salt‐sensitive response (Boyd‐Shiwarski et al., 2020; Terker et al., 2015; Vallon et al., 2009; Vitzthum et al., 2014). It has also been reported that HS intake leads to an unexpected increase in phosphorylated NCC (pNCC, an activation marker) abundance in mice consuming a K^+^‐deficient diet (Terker et al., 2015). Therefore, we next quantified NCC abundance in our animals and observed modestly lower abundances of phosphorylated and total NCC in HS‐fed animals compared with mice consuming NS (Figure 3b–d). The phosphorylated‐to‐total NCC ratio was not different between groups (Figure 3e). NCC is activated following direct phosphorylation by the kinases SPAK and OxSR1. We also observed reduced phosphorylated abundances of these kinases and total OxSR1 abundance (Figure 3b,f,h). Differences in total SPAK abundance were not detected between groups (Figure 3b,g).

**FIGURE 4:**
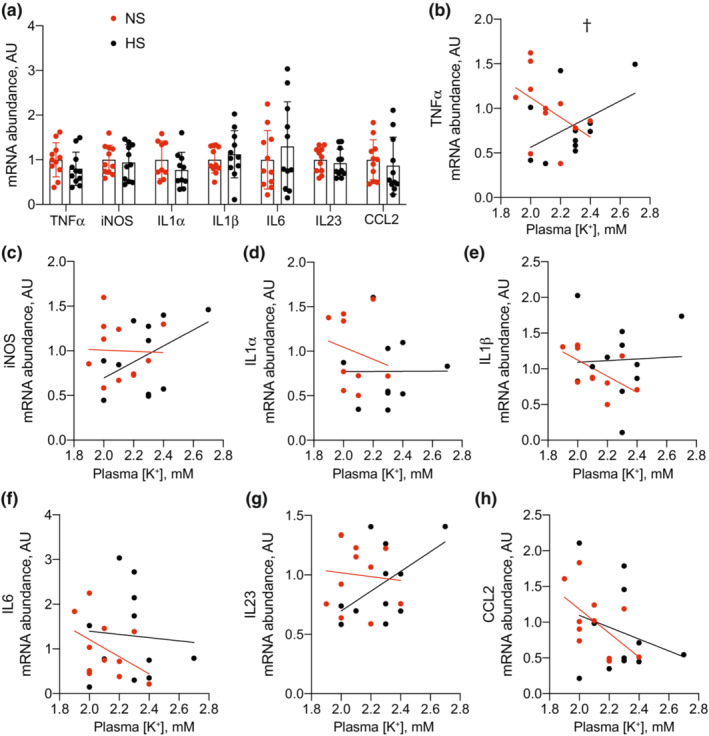
Total kidney transcript abundance of proinflammatory transcripts in mice consuming NS and HS diets. (a) Total kidney proinflammatory transcript abundance from NS and HS‐fed animals. (b–h) Linear regression analyses demonstrating the relationship between each transcript and blood K^+^. *N* = 11 per group. ^†^ *p* < 0.05 for the difference in slopes between lines for each group. HS, high salt; NS, normal salt.

**FIGURE 5:**
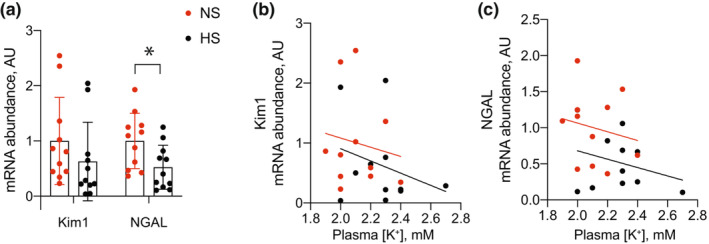
Total kidney transcript abundance of injury transcripts in mice consuming NS and HS diets. (a) Total kidney injury transcript abundance from NS and HS‐fed animals. (b, c) Linear regression analyses demonstrating the relationship between each transcript and blood K^+^. *N* = 11 per group. *p < 0.05 by unpaired *t*‐test corrected for multiple comparisons using Bonferroni correction. HS, high salt; NS, normal salt.

**FIGURE 6:**
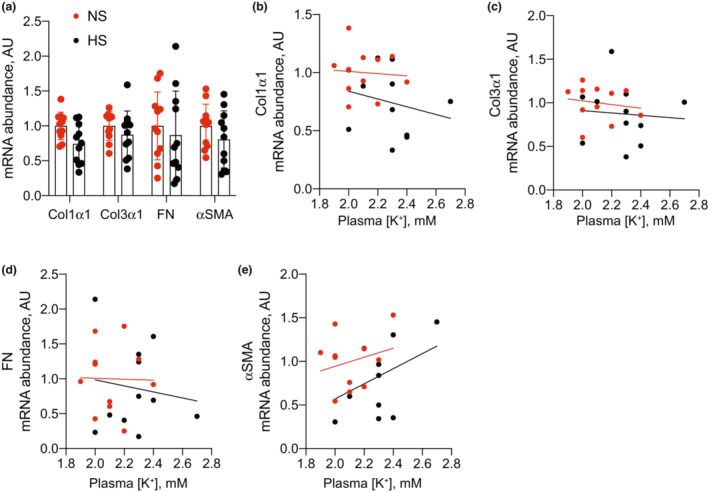
Total kidney transcript abundance of fibrosis transcripts in mice consuming NS and HS diets. (a) Total kidney fibrosis transcript abundance from NS and HS‐fed animals. (b–e) Linear regression analyses demonstrating the relationship between each transcript and blood K^+^. *N* = 11 per group. HS, high salt; NS, normal salt.

To assess pathological kidney effects, we subsequently measured mRNA abundance of inflammatory, injury, and fibrosis markers. Total kidney abundance of the proinflammatory transcripts TNFα, iNOS, IL1α, IL1β, IL6, IL23, and CCL2 were not different between groups (Figure 4a). After performing linear regression analyses to determine the relationship between each inflammatory marker and blood K^+^, we observed dietary Na^+^ content did not alter the relationship for any of the markers except TNFα, where HS was determined to reduce the influence of low blood K^+^ on increased TNFα mRNA abundance (Figure 4b–h). Abundance of the injury markers Kim1 and NGAL both trended lower in the HS group, but only differences in NGAL reached statistical significance (Figure 5a). Linear regression analyses did not reveal HS to have any effect on the relationship between injury markers and blood K^+^ (Figure 5b,c). Similarly, we did not observe major differences in fibrosis markers. Although Col1α1 trended lower in the HS group, it did not meet the threshold for statistical significance after correction for multiple comparisons (Figure 6a). Col3α1, FN, and αSMA remained unchanged (Figure 6a). Similar to other transcripts already discussed, dietary Na^+^ intake did not alter the relationship between fibrosis markers and blood K^+^ levels (Figure 6b–e).

## 4 DISCUSSION

Increased dietary Na^+^ is considered a significant contributor to high rates of hypertension and poor cardiovascular outcomes. Low dietary K^+^ also contributes to these same negative outcomes. Studies aimed at understanding mechanistic relationships between Na^+^, K^+^, and cardiovascular disease use modified animal diets that have been adjusted for one or both of these micronutrients to test hypotheses. We recently reported that a diet high in Na^+^ and low in K^+^ causes kidney‐specific inflammation, injury, and fibrosis and that the low dietary K^+^ is essential for deleterious effects. Low K^+^ alone was adequate for this response, while HS in isolation was not when compared to mice consuming a normal diet. However, it remained unclear what effect, if any, the addition of HS has in this model. Therefore, we tested effects of adding HS to a low K^+^ diet. Here we have demonstrated that while HS raised blood pressure in this context, it did not increase the renal abundance of proinflammatory cytokines, injury, or fibrosis transcripts. Additionally, it did not enhance the phosphorylation of NCC or its upstream regulatory kinases, which are known to be stimulated by low K^+^.

Results suggest that reduced K^+^ is the dominant influence on the observed kidney phenotype in animal models that use a combination of HS and low K^+^ diets. Because HS intake is known to increase distal K^+^ secretion and can lower blood K^+^ (Young, 1985; Young et al., 1984), we wanted to avoid Na^+^‐induced K^+^ reductions in our experiment as a potential confounder. For this reason, we added a minimal amount (0.04%) of dietary K^+^ to the HS‐consuming animals in an attempt to match blood K^+^ levels in our two groups. While the blood K^+^ values were similar, they were slightly higher in the animals consuming HS. This slightly higher blood K^+^ may account for the modestly reduced NGAL transcript abundance as well as pNCC, total NCC, OxSR1, and pSPAK/pOxSR1 abundances. We have previously demonstrated that these variables adopt a negative linear, or hyperbolic, association with blood K^+^ (Terker et al., 2022; Terker, Yarbrough, et al., 2016; Terker, Zhang, et al., 2016). Therefore, even slightly higher blood K^+^, as observed in the HS group, could underlie the detected differences. While on normal K^+^ it is thought that increased Na^+^ intake reduces pNCC (Vallon et al., 2009), we have previously demonstrated that high Na^+^ added to a K^+^‐deficient diet increases pNCC (Terker et al., 2015). Our current results, which have controlled for blood K^+^ suggest those prior data are likely reflective of a Na^+^‐induced reduction in blood K^+^ and increased activation of NCC following the addition of HS.

Overall our data provide evidence that high Na^+^ intake, independent of blood K^+^, does not worsen any of the measured parameters. This is further supported by our linear regression analyses which demonstrate the addition of HS does not affect the relationship between transcript abundance and blood K^+^ for nearly all tested parameters. This does not imply that high dietary Na^+^ cannot worsen kidney or cardiovascular health. There is an abundance of data suggesting it does and our results should not be interpreted as contradicting this body of literature. Our study highlights several important ways that Na^+^ can have a negative impact. First, we have reproduced known hypertensive effects of high Na^+^ intake in low K^+^ states. This is largely thought to be a result of increased NCC‐mediated Na^+^ reabsorption, although proximal transport pathways likely contribute as well. Increased NCC activity in a low K^+^ state is thought to limit distal Na^+^ delivery and reduce K^+^ secretion in principal cells (Ellison & Welling, 2021). In this setting, the cost of defending K^+^ homeostasis is increased Na^+^ reabsorption and higher blood pressure. While effects of higher blood pressure to the extent observed in this study are harmful over the long‐term (SPRINT Research Group et al., 2015), our study was likely too short to detect such effects. Second, as already mentioned, high Na^+^ intake can reduce blood K^+^ (Young, 1985; Young et al., 1984). While we sought to isolate effects of Na^+^ by matching blood K^+^ between groups, high Na^+^ intake can lower blood K^+^ across all levels of K^+^ consumption and therefore will worsen the observed phenotype in a K^+^‐dependent manner. It should also be appreciated that our conclusions apply specifically to the case of reduced dietary K^+^. Whether K^+^‐independent effects of increased Na^+^ consumption exist in other scenarios with reduced blood K^+^, such as aldosterone or angiotensin II infusion, remains unanswered. Substantial work to understand the mechanisms of hypertension has been performed using angiotensin II infusion coupled with high salt feeding. These studies point to an important role of immune cell activation in the pathogenesis (Guzik et al., 2007; Xiao & Harrison, 2020). We have previously demonstrated a dense immune cell infiltration after low K^+^ feeding (Terker et al., 2022). Whether there is an overlap between these two models remains an unanswered question. Angiotensin II infusion stimulates aldosterone release and primes animals for K^+^ depletion (Veiras et al., 2016). Whether K^+^ loss underlies some of the immune cell activations in models of angiotensin II infusion remains unclear and future studies should address this issue.

Recommendations for dietary Na^+^ intake have been published and periodically revised for years. While guidelines for K^+^ are not recognized as widely, they are published (USDA, 2020), though we are still trying to understand the optimal intake for both. Currently, clinical trials are attempting to identify optimal K^+^ intake for those with CKD (Gritter et al., 2022). We also do not fully appreciate how these cations interact with each other to amplify or minimize harmful effects. Such information is especially important in the prevention and management of CKD where dietary counseling is a pillar of patient care. Considerable time and effort are spent counseling patients on what to eat both as a primary preventive measure and to slow disease progression. Absent accurate information underlying these recommendations, such efforts will be largely ineffective. Our results highlight the importance of determining optimal dietary consumption of Na^+^ and K^+^ in both healthy individuals and those with chronic illnesses.

### 4.1 Limitations

The lack of a difference in weight change throughout the study period suggests overall food and water intake were similar, although we did not measure intake directly. Additionally, we did not measure 24 h urine volume. Low K^+^ intake is known to cause nephrogenic diabetes insipidus in mice (Al‐Qusairi et al., 2021), and there could have been a difference in polyuria that affected blood pressure in our animals. Urine creatinine was reduced in the HS group suggesting increased urine output, but this will need to be determined in future studies. We did not report aldosterone measurements precluding conclusions regarding its role in our observed phenotype. Lastly, we did not include groups that were treated with a normal diet or high salt diet with normal K^+^. These measurements were included in our previous report (Terker et al., 2022) and demonstrated that high salt alone did not increase inflammatory, injury, or fibrosis markers in kidney. Because these groups were not also included in the current work we are unable to draw direct comparisons, although our data in aggregate suggest transcript abundance is much higher in the two groups reported here relative to normal diet and high salt/normal K^+^‐treated animals.

## CONFLICT OF INTEREST STATEMENT

The authors have no conflicts of interest to declare.

## ETHICS STATEMENT

All animal experiments were performed in accordance within the guidelines and with the approval of the Institutional Animal Care and Use Committee of Vanderbilt University Medical Center.
